# CHANGING NEEDS AND NEEDS ASSESSMENT IN THE TRANSITION BETWEEN DISABILITY AND OLD AGE

**DOI:** 10.1093/geroni/igz038.1280

**Published:** 2019-11-08

**Authors:** Tine Rostgaard, Lea Graff

**Affiliations:** 1 VIVE - The Danish Center for Social Science Research, Copenhagen, Denmark; 2 VIVE, Copenhagen, Hovedstaden, Denmark

## Abstract

As our societies age, we see more people with disabilities living well into old age. However, there are different societal, systemic and individual assumptions about needs, rights and obligations associated with frail older people and people with disabilities. The paper presents quantitative results from a Danish study investigating what challenges ageing of society pose for the individual as well as for the welfare state in regards to meeting the needs of those who either age into disability or age with disability. Using panel data from the Danish Level of Living Survey from 1997-2017, we investigate how ADL related needs for care have changed for the 52+ year olds and we project how needs will change in the near future. Finally, we show how different systemic approaches to need assessment for those under or above 65, but with otherwise identical and socio-economic backgrounds, result in a very different service utility.

